# Degradation of Zearalenone by Dielectric Barrier Discharge Cold Plasma and Its Effect on Maize Quality

**DOI:** 10.3390/foods12061129

**Published:** 2023-03-07

**Authors:** Zhe Zheng, Liyang Niu, Wencheng Yang, Yi Chen, Yousheng Huang, Chang Li

**Affiliations:** 1 State Key Laboratory of Food Science and Technology, China-Canada Joint Laboratory of Food Science and Technology (Nanchang), Key Laboratory of Bioactive Polysaccharides of Jiangxi Province, Nanchang University, Nanchang 330047, China; 2 Jiangxi Institute of Analysis and Testing, Nanchang 330029, China; 3 Institute of Development and Research, Jiangxi General Institute of Testing and Certification, Nanchang 330029, China

**Keywords:** cold plasma, zearalenone, degradation, maize, quality, food safety

## Abstract

In this study, a dielectric barrier discharge (DBD) cold plasma was used to degrade zearalenone, and the degradation efficiency and the quality of maize were evaluated. The results showed that the zearalenone degradation rates increased with the increase in voltage and time. When it was treated at 50 KV for 120 s, the degradation percentage of the zearalenone in maize could reach 56.57%. The kinetics’ analysis showed that the degradation followed a first-order reaction. The crude fiber of the maize reduced after the cold plasma treatment. In addition, cold plasma treatment did not significantly change the crude protein content, but slightly changed the fatty acid and color. The changes in maize quality are generally acceptable. DBD cold plasma may be a promising approach to reducing zearalenone in maize.

## 1. Introduction

Mycotoxins are toxic secondary metabolites with low molecular weight produced by fungi that often contaminate grains during storage, which severely influence food safety. Zearalenone is a nonsteroidal estrogenic mycotoxin produced by the Fusarium species [1,2]. Zearalenone has been detected in many grains, but it is most commonly found in maize [3]. The ingestion of foods contaminated by zearalenone can raise the levels of estrogen in humans, posing a serious health risk [4]. In vivo, zearalenone can participate directly in and interfere with the reproductive process, leading to cell and DNA damage [5]. Of particular concern is the fact that zearalenone can accumulate after entering the food chain, where it has received much attention for its threat to food safety. Therefore, zearalenone degradation has become a research topic of interest in recent years.

A number of strategies have been proposed to reduce the amount of zearalenone in grains, and these mainly include physical, chemical, and biological approaches [6]. These approaches have been investigated in several studies, and each has achieved some degree of success in terms of its efficiency in degrading zearalenone, but most of them have some drawbacks, including poor efficiency, reduced nutritional value, and less palatability of the feed [7,8]. Traditional physical methods have been used to degrade mycotoxins, but most of them have unstable effects and require large-scale, expensive equipment [5]. Chemical methods may decrease the nutritional quality of food and lead to chemical residues [9]. Biological methods require harsh conditions and are relatively expensive, making them unsuitable for large-scale food processing [10]. Therefore, it is imperative to seek new effective degradation strategies.

In this study, we proposed a method of decontamination using novel non-thermal food-processing techniques to degrade zearalenone in maize. Cold plasma, as a new non-thermal food processing technology, has received much attention in recent years. Cold plasma is a kind of partially or wholly ionized state of gas generally generated by gas discharges, which consists of electrons, ions, free radicals, and excited particles [11,12]. Cold plasma has been used in degrading mycotoxins, such as aflatoxin, alternariol, deoxynivalenol, etc. [13,14,15,16]. Cold plasma has the advantages of short treatment time, good degradation efficiency, and high safety compared with the conventional technologies [17,18]. The previous studies have confirmed that cold plasma can effectively degrade zearalenone standard [19,20]. However, the grain quality is also of great concern when it is subject to cold plasma treatment. Whether cold plasma has the same degradation effect on the zearalenone spiked in maize and how the maize quality changes after the treatment with cold plasma has not been reported in the existing studies. 

Herein, we assessed the feasibility of zearalenone degradation via cold plasma. The zearalenone standard was spiked in maize kernels to mimic contamination. The effect of cold plasma treatment on the quality of maize was investigated, together with the effect of cold plasma on the degradation of zearalenone spiked in maize.

## 2. Materials and Methods

### 2.1. Materials

Zearalenone standard in acetonitrile (50 mg/L) was purchased from ANPEL (ANPEL Laboratory Technologies Co., Ltd., Shanghai, China). Other reagents were purchased from Aladdin (Aladdin Biochemical Technology Co., Shanghai, China), which were of analytical grade and used without further purification. Zearalenone standard was diluted in acetonitrile to obtain a concentration of 50 ng/mL and stored at −20 °C. The maize was purchased from the local market. The maize was confirmed to be free of zearalenone contamination by LC-MS/MS assay.

### 2.2. Experimental Apparatus

**Figure 1:**
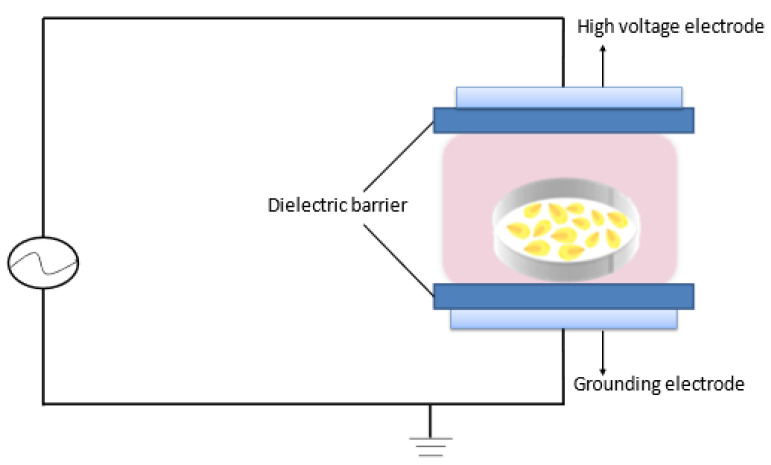
Schematic diagram of cold plasma.

The cold plasma generator was assembled by a high voltage transformer, a controller, and a pair of discharge electrodes. Figure 1 presents a schematic diagram of the DBD plasma generator used in the present study. The work gas is atmospheric air.

### 2.3. Conditions for the Treatment of Maize by Cold Plasma

The contaminated sample was prepared referring to the methods by Feizollahi [21] with a minor modification. Briefly, the maize kernels free of zearalenone were placed in a petri dish. Five milliliters of zearalenone standard solution were added to soak the maize kernels for 2 min. The maize kernels were then air-dried in a fume hood before being exposed to the cold plasma. 

The samples were treated by cold plasma at different voltages (30, 40, and 50 KV) for different times (10, 30, 60, 90, and 120 s). Two PVC plates of 2 mm thickness were chosen for the dielectric material, and the distance between these two parallel plates was fixed at 2 cm. After the cold plasma treatment, the maize kernels were soaked in 50% (*v/v*) acetonitrile water solution for 30 min. The standard was redissolved in 50% (*v/v*) acetonitrile water after the treatment. Each treatment was performed on three sample replicates.

### 2.4. Zearalenone Determination

Zearalenone was quantified with LC-MS/MS (Agilent Technologies, Santa Clara, CA, USA), according to the previous method [19]. Zearalenone standard solution was diluted in acetonitrile and the standard curves were established at concentrations of 1, 5, 10, 25, and 50 ng/mL, respectively. All chromatographic separations were performed using a C_18_ column (2.1 × 50 mm, 1.8 µm); the mobile phase was (A) acetonitrile (*v/v*) and (B) 0.1% formic acid in water (*v/v*). The sample injection volume was 5 µL. Isocratic elution was performed with phases A and B at a ratio of 10:90 for 9 min. The flow rate was 0.3 mL/min. The UPLC system was coupled to triple quadrupole mass spectrometer with electrospray ion source (ESI) and the detection was performed in positive ionization mode. The equation of the standard curve was used to calculate the concentration of the zearalenone.

### 2.5. Degradation Kinetics of Zearalenone

In this study, the degradation kinetics of zearalenone by DBD cold plasma was investigated. The degradation percentage (y) was used as the vertical coordinate and the reaction time (x) was used as the horizontal coordinate to draw a scatter plot. Kinetic fits were performed using curve estimates from the SPSS software, fitting logarithmic, quadratic, cubic and power functions, respectively. The effect of treatment time on the kinetics of zearalenone degradation was evaluated. The function model with the best fitting effect was selected. In addition, the relevant parameters of the kinetic equation were calculated.

### 2.6. Analysis of Maize Color

A colorimeter was used to analyze the surface color of maize. The instrument was calibrated firstly with a calibration standard white plate and the color of the maize was analyzed using the Commission International Eclairage (CIE)-L *, a *, b * colorimetric system. Several specific points of a few maize kernels were selected for colorimetric analysis.

### 2.7. Determination of Fatty Acid

The fatty acid content of maize was determined according to the Chinese National Standard Method GB/T20570-2015 [22]. Briefly, 10 g of the maize powder and 50 mL anhydrous ethanol were mixed in a conical flask. The mixture was shaken for 30 min and allowed to stand before filtering into a colorimetric tube. The filtrate was added to a conical flask to which ultra-pure water had been added. Then, the filtrate was added with 5 drops of phenolphthalein indicator and titrated with KOH (0.5 mol/L) standard solution until the color was reddish and did not fade within 30 s.

The fatty acid content was calculated by Equation (1):

(1) $$A_{k}=\left(V_{1}-V_{0}\right)\times c\times 56.1\times \frac{50}{25}\times \frac{100}{m\left(100-\omega \right)}\times 100$$

*A_k_*: The mass of fatty acids (mg/100 g).
*V*_1_: The volume of KOH consumed by titration of the sample (mL).
*V*_0_: The volume of KOH consumed by titration of the control sample (mL).
*c*: The concentration of KOH standard titration solution (mol/L).
*m*: The mass of sample (g).
*ω*: The moisture content in 100 g sample (g).

### 2.8. Determination of Crude Protein

The crude protein content (g/100 g) of the samples was obtained by using the Kjeldahl method following the Chinese National Standard Method GB/T 5009.5-2016 [23] with a conversion factor of 6.25. Two grams of the maize powder were added to a Kjeldahl bottle to which 20 mL H_2_SO_4_ (0.05 mol/L), 0.2 g CuSO_4_, and 6 g K_2_SO_4_ had been added. The samples were digested until the light blue color became transparent and was left boiling for 1 h. The solution was allowed to cool down to room temperature and titrated until the color changed from purple to gray.

The protein content was calculated via Equation (2):

(2) $$X=\frac{\left(V_{1}-V_{2}\right)\times c\times 0.014}{m\times V_{3}/100}\times F\times 100$$

*X*: Crude protein content in the samples (g/100 g).
*V*_1_: The volume of H_2_SO_4_ consumed by titration of the sample (mL).
*V*_2_: The volume of H_2_SO_4_ consumed by titration of the control sample (mL).
*c*: The concentration of H_2_SO_4_ standard titration solution (mol/L).
*m*: The mass of sample (g).
*V*_3_: The volume of digestion solution (mL).
*F*: Conversion factor.

### 2.9. Determination of Crude Fiber

The crude fiber content was determined with the Chinese National Standard Method GB/T 5009.10-2003 [24]. Five grams of the maize powder was added to a conical flask to which boiled H_2_SO_4_ (1.25%) had been added. The solution was heated for 30 min and filtered with linen, and the residue was washed and transferred to another conical flask. Then, boiled NaOH (1.25%) was added to the flask. The insoluble residue was separated on a filter and washed. After drying and weighing, the loss of mass was analyzed via incineration.

The fiber content was calculated by Equation (3):

(3) $$X=\frac{G}{m}\times 100\%$$

*X*: Crude fiber content in the samples.
*G*: The mass of residue (g).
*m*: The mass of sample (g).

### 2.10. Statistical Analysis

Statistical analysis was carried out using SPSS 23.0, and a significant difference was verified by one-way ANOVA with Waller–Duncan’s multiple range test (*p* < 0.05).

## 3. Results and Discussion

### 3.1. Degradation Efficiency Analysis

**Figure 2:**
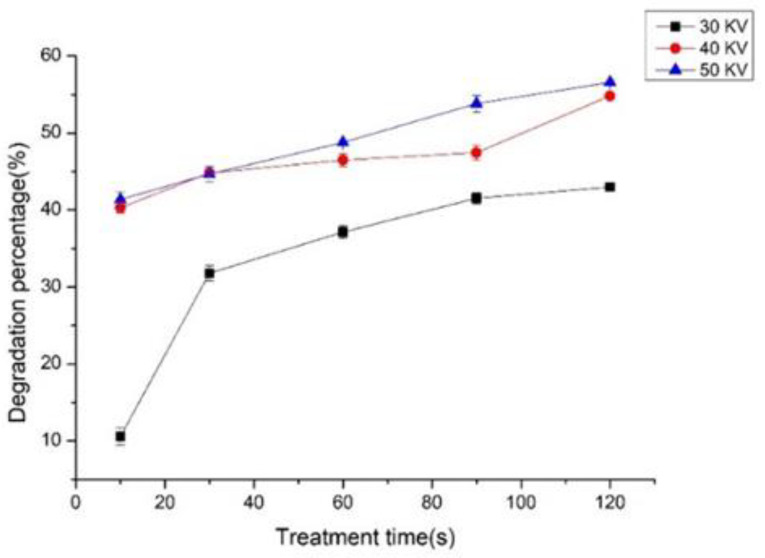
Voltage and time effects of DBD plasma on degradation percentage of zearalenone spiked in maize.

The effects of treatment time and voltage on the degradation of zearalenone were investigated. As shown in Figure 2, the degradation percentage of zearalenone increased with the treatment time and voltage. The contact time of the maize with the reactive species in the plasma increased with the treatment time, and as a result, the degradation percentage of zearalenone improved. With the voltage of 30 KV, zearalenone demonstrated a degradation of 10.57% for 10 s, 31.79% for 30 s, 37.14% for 60 s, 41.53% for 90 s, and 42.99% for 120 s, respectively. At this voltage, the generated cold plasma is lower in energy and less in reactive species’ amount. The degradation rate of zearalenone was accordingly lower accordingly. When the sample was treated for 120 s, zearalenone demonstrated a degradation of 42.99% at 30 KV, 54.82% at 40 KV, and 56.57% at 50 KV, respectively. High voltages can produce a higher number of radicals and charged particles in cold plasma [25,26,27]. Therefore, higher voltages are more effective for mycotoxin degradation, and voltage plays an important role in degradation efficiency.

**Figure 3:**
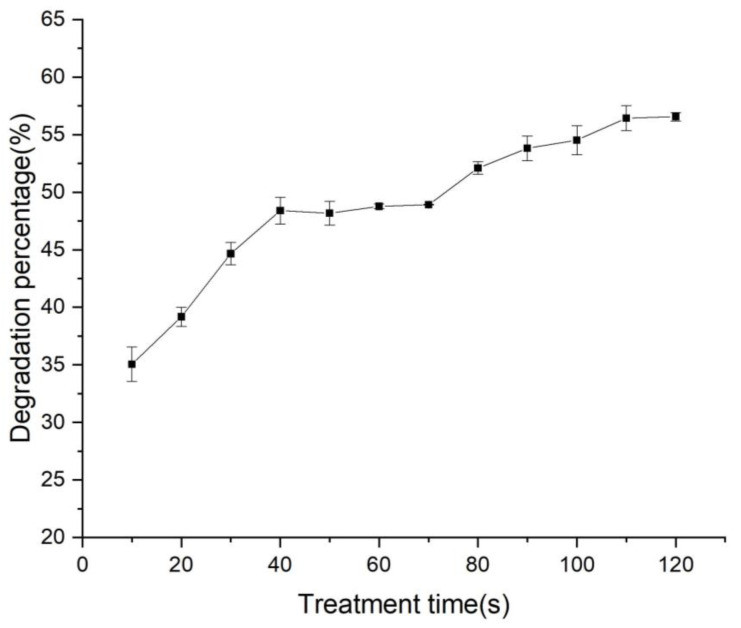
Degradation percentage of zearalenone treated at 50 KV.

As shown in Figure 3, the degradation rate gradually increases with the treatment time when the sample is treated at 50 KV. When the treatment time was extended to 120 s, the degradation percentage of zearalenone spiked in maize was 56.57%. Compared with the degradation result of the zearalenone in our previous work [19], the degradation percentage of the zearalenone spiked in maize was relatively lower. It could be attributed to the matrix effects of maize. Moreover, the penetration of the cold plasma is so weak that it only breaks down the zearalenone on the surface of the maize, leaving the zearalenone in the maize untouched. In a previous report, Sebaei et al. used γ-ray to degrade zearalenone in twelve samples that were artificially contaminated. The authors found that at 20 kGy, the reductions were 51.1% in yellow corn samples [28]. In addition, Yang et al. investigated zearalenone removal efficiency by comparing the concentrations of zearalenone in the reaction solution and initial solution, they found that about 50% of zearalenone was photocatalytic degraded within 90 min over the Bi_2_WO_6_ in the corn oil [29]. By comparing with the previous studies, our study achieved an acceptable degradation rate in a shorter treatment time. Although we have not yet conducted experiments on naturally contaminated maize, based on the present results, we believe that the cold plasma shows some potential in the degradation process of zearalenone, compared with other methods. We will conduct in-depth studies on naturally contaminated maize in the future. Further evaluation of the application of cold plasma to a large number of naturally contaminated samples is the critical next step for scaling-up.

### 3.2. Degradation Kinetics Analysis

The previous study confirmed that the degradation process of zearalenone standard is in accordance with the first-order kinetic degradation [19]. Similarly, in the present study, the degradation of zearalenone spiked in maize by plasma treatment was also investigated from a kinetic point of view, in order to investigate the effect of maize matrix itself on the degradation of zearalenone. First-order kinetics fits were performed using the SPSS software, fitting logarithmic, quadratic, cubic, and power function models, respectively. The kinetic model parameters and correlation coefficients for zearalenone degradation are given in Table 1. Compared with the previous study [19], the kinetics of zearalenone in maize are different from those of the zearalenone standard. It is possible that the rate or mode of degradation of zearalenone was altered due to the effect of the maize matrix.

**Figure 4:**
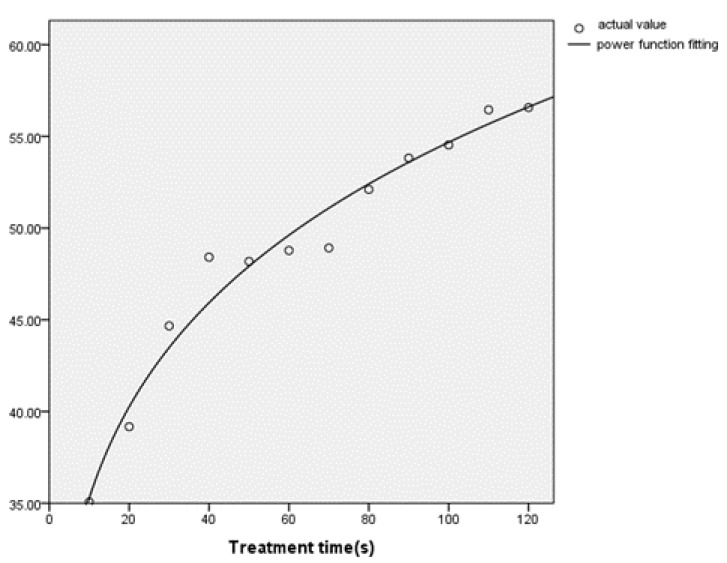
Effect of reaction time on degradation of zearalenone spiked in maize.

The determination of kinetic model mainly refers to its correlation coefficient. The closer the correlation coefficient is to 1, the more likely the model can reflect the real kinetics. The correlation coefficient of the power function R² = 0.971 is closer to 1, so it can be selected as the degradation kinetics’ model of zearalenone in maize. The expression is y = 22.766 (x^0.19^), and the fitting effect is shown in Figure 4. The degradation of zearalenone is accorded with the first-order kinetics.

### 3.3. Effect of Cold Plasma Treatment on Maize Color

The effect of different plasma parameters on the color of the maize is shown in Table 2. L *, a * and b * values represent brightness, redness, and yellowness, respectively. From Table 2, it can be seen that the L * values decreased, a * values did not change significantly and b * values increased after cold plasma treatment. The maize treated with the cold plasma was a darker yellow in general color. This may be due to changes in zeaxanthin, carotene, and lutein in the maize skin and may be considered as a subject for future research. Cold plasma with air as working gas can generate ROS and RNS, which can destroy the chemical structure of pigments susceptible to oxidation in food. Wang et al. [30] found that cold plasma treatment had a negative effect on beef color, with an increase in b * values. Xie et al. [31] subjected fresh-cut kiwifruit to cold plasma treatment, and found that L * values decreased, a * and b * values increased after treatment; however, the changes may be due to the fact that kiwifruit is freshly cut and then oxidized by the reactive species in the plasma, leading to the degradation in chlorophyll and carotenoids, which in turn darkens the color of the fruit. In conclusion, the change in maize color is related to the treatment parameters of cold plasma and the pigments in maize, and further research is needed on the optimal treatment parameters, for example, using argon as working gas, to avoid the color change in the context of ensuring a high degradation percentage.

### 3.4. Effect of Cold Plasma Treatment on the Fatty Acid of Maize

The fatty acid content, crude fiber content, and crude protein content determined the quality of maize during processing and storage. When the free fatty acid increased, it was often considered as a deteriorative change in the quality of the maize [32,33]. 

In order to investigate the effects of cold plasma on the storage quality in maize, the changes in fatty acid in maize were determined pre- and post-treatment by cold plasma and the results are shown in Table 3. As the treatment time and the voltage increased, the fatty acid content in the maize showed an overall trend of increasing, with a significant increase especially under the treatment condition at 50 KV for 120 s. The reactive species generated in cold plasma have strong oxidizing properties, which can lead to the oxidation of lipids [34]. In addition, these reactive species with oxidative properties also accelerate the direct decomposition of lipids, thus the fatty acid content of maize increases after the treatment. However, according to GB/T20570-2015, the content of free fatty acid in the maize did not reach the level of slight unsuitability for storage (<78 mg KOH/100 g dry basis). It indicates that the rise in the fatty acids in the maize after the cold plasma treatment are acceptable in terms of quality standards.

### 3.5. Effect of Cold Plasma Treatment on the Crude Protein and Crude Fiber of Maize

The crude fiber and the crude protein content are usually used as conventional indicators to evaluate the variation in the nutrient content of maize [35]. In order to investigate the effects of cold plasma on the nutrients in maize, the changes in crude fiber and crude protein content in maize were determined pre- and post-treatment by cold plasma and the results are shown in Table 3. The crude fiber content of maize did not change significantly with lower treatment voltage or shorter treatment time. However, as the treatment time or the voltage increased, the content of crude fiber in the maize showed a decreasing trend. It may be attributed to the breakdown of fiber through the interaction with high-energy species [36,37]. 

Moreover, after the cold plasma treatment, there was no significant change in the content of crude protein in the maize. This may be due to the fact that the penetration of the cold plasma is not strong, it only acts on the surface of the maize, and has a small effect on the protein under epidermis. The protein is mostly distributed between the endosperm and the germ; meanwhile, the coarse fiber is a characteristic composition of the epidermis [38]. In general, when maize was used as feed, the lower fiber content and higher protein content were often considered to have better palatability [39]. From the results, the effect of cold plasma treatment on the quality of maize is generally acceptable, and it can even improve the quality in some aspects. It suggests that cold plasma has an application potential in the processing and storage of maize.

## 4. Conclusions

The effects of cold plasma on the zearalenone degradation and the quality of maize were elucidated through this research. The treatment time and voltage are the main influencing factors of cold plasma on the degradation of zearalenone and the changes in maize quality. The degradation percentage of zearalenone in the maize increased, as the treatment time was extended or the voltage was boosted. The matrix of maize may affect the degradation of zearalenone. The fatty acid in maize increased after cold plasma treatment, but the values did not reach the level of slight unsuitability for storage. The crude fiber content of maize decreased slightly after cold plasma treatment, and there was no significant change in crude protein content. The quality changes are generally acceptable. Therefore, the cold plasma presents a potential application in the reduction in zearalenone in maize.

## Figures and Tables

**Table 1:** Parameters and R² value of reaction kinetic model of zearalenone degradation.

Model	Equation	F	R²	Sig
Log function	y = 8.658 logx + 14.531	301.713	0.968	<0.001
Quadratic function	y = 33.463 + 0.349x − 0.01x²	80.802	0.947	<0.001
Cubic function	y = 0.677x − 0.07x² + 3.109 × 10^−5^x^3^ + 29.219	85.985	0.970	<0.001
Power function	y = 22.766 (x^0.19^)	338.234	0.971	<0.001

**Table 2:** Effect of cold plasma treatment on the surface color of maize.

Treatment Voltage (KV)	Treatment Time (s)	L *	a *	b *
/	0	28.980 ± 2.587c	2.65 ± 2.913 abc	19.854 ± 2.124 a
30 KV	10	20.094 ± 2.360 ab	3.592 ± 1.522 bc	28.002 ± 4.756 b
	30	19.544 ± 1.392 ab	3.14 ± 2.562 abc	33.566 ± 4.671 bc
	60	21.734 ± 3.217 b	3.69 ± 0.767 bc	32.426 ± 2.293 bc
	90	22.314 ± 3.338 b	4.002 ± 0.807 bc	33.088 ± 2.169 bc
	120	18.354 ± 2.758 ab	2.17 ± 1.808 abc	32.472 ± 4.545 bc
40 KV	10	18.146 ± 2.492 ab	1.156 ± 1.128 ab	32.446 ± 4.074 bc
	30	15.822 ± 1.276 a	0.174 ± 1.696 a	31.952 ± 2.576 bc
	60	19.242 ± 3.225 ab	1.312 ± 1.846 ab	31.940 ± 3.591 bc
	90	18.152 ± 3.327 ab	0.852 ± 2.021 ab	30.756 ± 4.829 bc
	120	18.452 ± 1.510 ab	2.24 ± 2.055 abc	32.956 ± 2.846 bc
50 KV	10	19.456 ± 3.000 ab	3.488 ± 1.962 bc	32.534 ± 4.542 bc
	30	21.302 ± 4.481 b	4.938 ± 2.153 c	34.520 ± 5.535 bc
	60	18.756 ± 2.862 ab	3.704 ± 1.341 bc	33.166 ± 3.779 bc
	90	22.954 ± 5.193 b	3.972 ± 2.069 bc	31.374 ± 5.549 bc
	120	18.918 ± 2.052 ab	0.186 ± 2.161 a	35.136 ± 1.426 c

Data labeled with different letters or combinations of letters means significant differences (*p* < 0.05).* indicates that L, a, and b are based on the CIE system to distinguish them from the Hunter system.

**Table 3:** Effects of cold plasma treatment on the nutrients of maize.

Treatment Voltage (KV)	Treatment Time (s)	Fatty Acids (mg/100 g)	Crude Fiber (%)	Crude Protein (%)
/	0	64.35 ± 1.05 a	2.30 ± 0 c	9.18 ± 0.17 a
30	10	65.35 ± 2.6 abc	2.15 ± 0.05 c	9.05 ± 0.07 a
	30	68.4 ± 0.9 abc	2.1 ± 0 c	8.875 ± 0.095 a
	60	66.55 ± 0.45 abc	2 ± 0.1 abc	8.9 ± 0.13 a
	90	65.7 ± 1.5 abc	1.95 ± 0.45 abc	9.155 ± 0.015 a
	120	65.1 ± 3.4 ab	1.95 ± 0.15 abc	9.21 ± 0.25 a
40	10	66.7 ± 2.6 abc	2.05 ± 0.15 bc	9.16 ± 0 a
	30	67.5 ± 0.9 abc	1.75 ± 0.15 abc	8.92 ± 0.05 a
	60	68.15 ± 0.45 abc	1.7 ± 0.2 abc	9.16 ± 0.21 a
	90	67.4 ± 1.5 abc	1.7 ± 0 abc	9 ± 0.22 a
	120	69.3 ± 3.4 abc	1.55 ± 0.15 ab	8.975 ± 0.035 a
50	10	66.15 ± 2.55 abc	1.7 ± 0.1 abc	9.065 ± 0.085 a
	30	66.8 ± 0.1 abc	1.65 ± 0.05 abc	9.08 ± 0.14 a
	60	69.3 ± 0.2 abc	1.8 ± 0.05 abc	9.105 ± 0.105 a
	90	69.55 ± 0.95 bc	1.65 ± 0.15 abc	8.995 ± 0.035 a
	120	70.3 ± 1.2 c	1.5 ± 0 a	9.14 ± 0 a

Data labeled with different letters or combinations of letters means significant differences (*p* < 0.05). The fatty acids were expressed of KOH required to neutralise the free fatty acids in 100 g of dry basis in mg/100 g.

## Data Availability

The datasets generated during and/or analyzed during the current study are available from the corresponding author on reasonable request.
